# Bacterial and Fungal Microbiota Associated with the Ensiling of Wet Soybean Curd Residue under Prompt and Delayed Sealing Conditions

**DOI:** 10.3390/microorganisms8091334

**Published:** 2020-09-01

**Authors:** Ajmal Wali, Naoki Nishino

**Affiliations:** Graduate School of Environmental and Life Science, Okayama University, Okayama 700-8530, Japan; ajmal_hilal@yahoo.com

**Keywords:** amplicon sequencing, bacteria, fungi, silage, soybean curd residue

## Abstract

Wet soybean curd residue (SCR) obtained from two tofu factories (F1 and F2) was anaerobically stored with or without added beet pulp (BP). Sealing was performed on the day of tofu production (prompt sealing (PS)) or 2 days after SCR was piled and unprocessed (delayed sealing (DS)). Predominant lactic acid fermentation was observed regardless of the sealing time and BP addition. *Acinetobacter* spp. were the most abundant (>67%) bacteria in pre-ensiled SCR, regardless of the factory and sealing time. In PS silage, the abundances of typical lactic acid-producing bacteria, such as *Lactobacillus*, *Pediococcus*, and *Streptococcus* spp. reached >50%. In DS silage, *Acinetobacter* spp. were the most abundant in F1 products, whereas *Bacillus* spp. were the most abundant in long-stored F2 products. The fungal microbiota were highly diverse. Although *Candida*, *Aspergillus*, *Cladosporium*, *Hannaella*, and *Wallemia* spp. were found to be the most abundant fungal microbiota, no specific genera were associated with factory, sealing time, or fermentation products. These results indicated that owing to preceding processing, including heating, distinctive microbiota may have participated in the ensiling of wet by-products. Lactic acid fermentation was observed even in DS silage, and an association of *Bacillus* spp. was suggested.

## 1. Introduction

A large variety of wet by-products are produced by the food and beverage industries, with annual global amounts as high as one billion tons. Animal husbandry successfully makes use of these by-products as ingredients in animal feed [1]. The by-products are often stored under anaerobic fermentation in silos. Thus, ensiling is regarded as the most efficient way to preserve wet by-products. Furthermore, ensiled non-food biomass is also regarded as a renewable energy source, being used to produce biofuels.

A significant amount of soybean curd residue (SCR) is generated as a by-product from the manufacturing of tofu, a traditional Asian food prepared by coagulating soy milk. The sum of SCR in China, Korea, and Japan is approximately 3.9 million tons per year, equivalent to the annual amount of tomato pomace generated globally [2,3]. Although the low dry matter (DM) content (about 200 g/kg) of wet SCR poses a challenge to its handling and transportation, its high crude protein content (about 250 g/kg DM) makes it an attractive ingredient for use in animal feed. In addition, the storability of SCR can vary greatly between factories and seasons because of large differences in the handling procedures executed by vendors.

During tofu manufacturing, soybeans are milled and mashed. As a result, SCR is more easily compacted than other forage crops. A density of >900 kg/m^3^ is difficult to obtain in forage ensiling, even with fine chopping and intensive compaction. However, this can be easily achieved when using SCR, without the help of special devices [4]. Moreover, the physical properties of SCR may accelerate the establishment of an anaerobic environment, allowing for lactic acid fermentation without the need for additives. As a result of the heating step, during which the soy milk and SCR are separated, the resulting SCR can reach temperatures as high as 80 °C, which may restrict the general bacterial population at the 10^3–5^ CFU/g level and can change its bacterial composition under non-farm environmental conditions [4]. Meanwhile, the major components of soybean sugars are removed from soymilk during processing; hence, the addition of soluble sugars would help increase lactic acid fermentation of SCR silage. Because the moisture content of raw SCR is relatively high, the addition of dried by-products, such as wheat bran and beet pulp (BP), would also help improve the storability of SCR silage.

The industrial scale of tofu manufacturing varies widely across factories. In addition, their locations are scattered, and are separate from the livestock sector. Daily on-site ensiling is difficult to perform, thus sealing is often delayed in practice. This lowers the opportunity for the stable production of a high-quality feed and increases the risk of spoilage due to rancidity, especially in hot summers.

In this study, wet SCR was obtained from two tofu factories (F1 and F2) and ensiled with and without BP on the day of tofu production (prompt sealing (PS)) and 2 days after being piled and unprocessed (delayed sealing (DS)). Ensiling was performed for 6 months since the effects of DS could lead to changes in the microbiota under conditions of long-term storage. The objective was to characterize the microbiota associated with SCR silage stored under PS and DS conditions. Both bacterial and fungal microbiota were assessed using high-throughput amplicon sequencing.

## 2. Materials and Methods

### 2.1. Ensiling

Two sets of SCRs were obtained from F1 and F2, and each was further divided into two subsets. The first and second subsets of SCR were subjected to PS and DS ensiling, respectively. Briefly, 300 g of wet SCR was sealed in a plastic bag (Hiryu BN-12; Asahi Kasei Pax, Tokyo, Japan) in triplicate, with or without dried BP (60 g). Air was removed using a vacuum sealer (SQ-303; Asahi Kasei Pax, Tokyo, Japan), and the bags were stored at room temperature. The silos containing the samples were opened after 2 weeks and 3 and 6 months to examine the fermentation products and evaluate the bacterial and fungal microbiota.

### 2.2. Chemical Composition Analyses and Epiphytic Microbiota Counts

The DM content was determined after oven drying at 60 °C for 48 h. The pH values and fermentation products were determined using water extracts. The lactic acid, acetic acid, and ethanol contents were measured by ion-exclusion polymeric high-performance liquid chromatography with refractive index detection [5]. For the determination of soluble sugars, freeze-dried samples were extracted using an ethanol/water (80:20, *v*/*v*) solution. The fructose, glucose, sucrose, maltose, raffinose, and stachyose contents were determined by hydrophilic interaction chromatography with refractive index detection [6].

The levels of lactic acid bacteria, and yeast and molds were determined using de Man, Rogosa, Sharpe agar and Yeast Malt agar, respectively. The serially diluted plates were incubated at 30 °C for 2 days.

### 2.3. Microbiota Analyses

Silage samples were added to a 20× volume of sterilized phosphate-buffered saline (pH 7.4). DNA extraction was performed as described by Yu et al. [7]. DNA purification was performed using a commercial kit (DNeasy Blood & Tissue Kit; Qiagen, Germantown, MD, USA) according to the manufacturer’s instructions.

The resulting DNA was subjected to 2-step polymerase chain reaction (PCR) to generate amplicon libraries. For bacterial MiSeq analysis, primers targeting the V4 region of 16S rRNA genes (forward: 5′-ACACTCTTTCCCTACACGACGCTCTTCCGATCTGTGCCAGCMGCCGCGGTAA-3′; reverse: 5′-GTGACTGGAGTTCAGACGTGTGCTCTTCCGATCTGGACTACHVGGGTWTCTAAT-3′) were used in the first round PCR [8]. The PCR protocol was as follows: initiation at 94 °C for 2 min, followed by 25 cycles of 94 °C for 30 s, 50 °C for 30 s, and 72 °C for 30 s, and a final elongation at 72 °C for 5 min. For fungal MiSeq analysis, primers targeting the internal transcribed spacers (ITS2) separated by 5.8S (forward: 5′-ACACTCTTTCCCTACACGACGCTCTTCCGATCTGTGARTCATCGARTCTTTG-3′; reverse: 5′-GTGACTGGAGTTCAGACGTGTGCTCTTCCGATCTTCCTCCGCTTATTGATATGC-3′) were employed in the first round PCR [9]. The PCR protocol was as follows: initiation at 95 °C for 5 min, followed by 30 cycles of 95 °C for 30 s, 55 °C for 30 s, and 72 °C for 1 min, and a final elongation at 72 °C for 10 min. The products were purified using the Fast Gene Gel/PCR Extraction Kit (NIPPON Genetics Co., Ltd., Tokyo, Japan) and used in the second round PCR with the adapter-attached primers. The second PCR protocol was as follows: initiation at 94 °C for 2 min followed by 10 cycles of 94 °C for 30 s, 59 °C for 30 s, and 72 °C for 30 s, and a final elongation at 72 °C for 5 min. The PCR products were purified as described above.

### 2.4. Illumina MiSeq Sequencing

The purified amplicons were pair-end sequenced (2 × 250 bp) on an Illumina MiSeq platform at FASMAC Co., Ltd. (Kanagawa, Japan). The raw sequence data were analyzed using Quantitative Insights into Microbial Ecology (version 1.9.0). The 250-bp reads were truncated at any site, with an average quality score under 20. The truncated reads that were shorter than 225 bp were discarded. For primer matching, sequences with overlaps longer than 200 bp were assembled. The final reads obtained after pair-end joining were grouped into operational taxonomic units using a 97% similarity threshold. The sequence data were analyzed and categorized from the phylum to the family level using the default settings of the Ribosomal Database Project classifier. The results of the sequence analysis are available in the DDBJ Sequence Read Archive under project identification number PRJDB10470.

### 2.5. Data Analyses

The fermentation product data were subjected to two-way analysis of variance (ANOVA), with sealing time and BP addition as the main factors. The microbiota data were subjected to principal coordinate analysis (PCoA) to define the assignment and clustering accounting for the variations in the microbiota. Discriminant vectors with a Pearson correlation >0.7 were considered significant. Two-way ANOVA was performed using JMP software (version 11; SAS Institute, Tokyo, Japan), and PCoA was carried out using Primer (version 7) with the Permanova+ add-on (Primer-E; Plymouth Marine Laboratory, Plymouth, UK).

## 3. Results

### 3.1. Chemical Composition and Fermentation Products

The DM contents of raw SCR were 182 and 239 g/kg for the F1 and F2 products, respectively, indicating that the contents did not change much during piling and unprocessing for 2 days (Table 1). A small amount of lactic acid was observed in raw SCR, while acetic acid and NH_3_–N were found in DS pre-ensiled SCR.

When F1 SCR was ensiled, lactic acid fermentation was observed regardless of PS and DS (Table 2). The lactic acid levels were 37.5 and 55.5 g/kg DM at 2 weeks in PS and DS silage, respectively. After storage for 6 months, the lactic acid content increased by about 40% compared to that at 2 weeks. BP addition suppressed lactic acid production, and this effect was manifested in DS silage. The acetic acid content was 6–20% of the lactic acid content, with levels higher than PS silage. The addition of BP increased the acetic acid content in PS silage stored for 3 and 6 months. The ethanol content was lower than the acetic acid content and greater in DS than in PS silage. Although the NH_3_–N content was as low as 0.13 g/kg DM even in long-stored PS silage, the level was substantially high (as much as 2.0 g/kg DM) in DS silage. The addition of BP lowered the NH_3_–N level in DS silage but not in PS silage.

When F2 SCR was ensiled, lactic acid fermentation was predominant, and DS enhanced the lactic and acetic acid contents (Table 3). Similar to F1 SCR silages, BP addition suppressed the lactic acid content in DS silage and increased the acetic acid content in PS silage. Likewise, the NH_3_–N content attained at 2.0 g/kg DM in DS silage and BP addition only lowered the NH_3_–N levels in PS silage. Unlike F1 SCR ensiling, however, substantial amounts of propionic and butyric acid were produced, and the levels of 20 g/kg DM at 6 months were greater than those of acetic acid in PS silage.

### 3.2. Bacterial Microbiota

**Figure 1:**
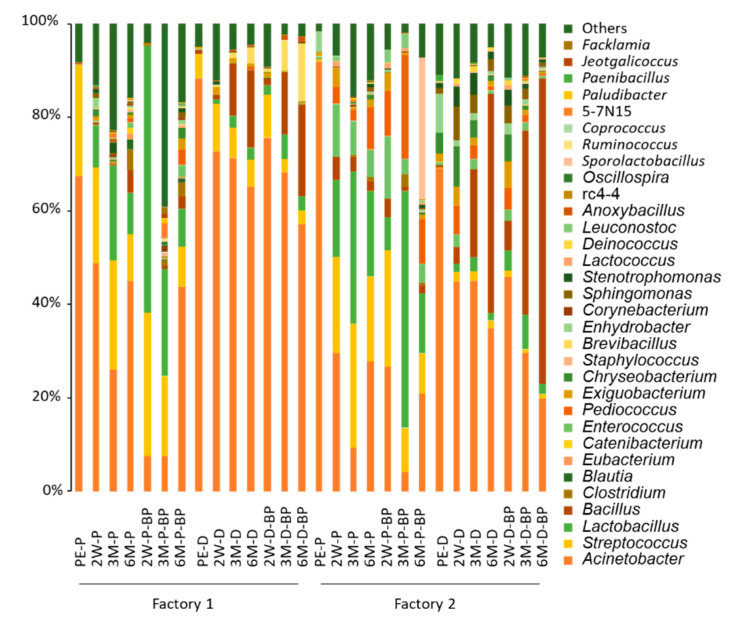
Genus-level bacterial microbiota in soybean curd residue silage prepared with prompt and delayed sealing and with and without beet pulp addition. PE, 2W, 3M, and 6M indicate pre-ensiled material and silage stored for 2 weeks, 3 months, and 6 months, respectively. P, D, and BP after the hyphenation denote prompt sealing, delayed sealing, and beet pulp addition, respectively.

The MiSeq sequencing resulted in non-chimeric sequence reads with an average of 68,820 and 70,626 for F1 and F2 samples, respectively. *Acinetobacter* spp. were the most abundant bacteria (67.4%) in pre-ensiled F1 SCR (Figure 1). In F1 PS silages, the abundance of *Acinetobacter* spp. was 7.6–26.1% at 3 months, growing to >40% at 6 months. Although *Lactobacillus* spp. were undetectable in pre-ensiled F1 SCR, the abundance increased to 8.99% and >30% at 2 weeks and 3 months, respectively, in F1 PS silage. *Streptococcus* spp. were found at >20% in pre-ensiled F1 SCR. The abundance was retained until 3 months before decreasing to <10% at 6 months. After 6 months of storage, *Bacillus* (2.7–4.9%), *Enterococcus* (1.2–2.5%), and *Clostridium* spp. (3.0–4.4%) in F1 PS silage became non-negligible.

The abundance of *Acinetobacter* spp. in SCR increased to 88% during piling and unprocessing for 2 days. Although the abundance decreased during prolonged ensiling, levels of 57.2–65.2% were observed in F1 DS silage even after 6 months. BP addition did not affect the abundance of *Acinetobacter* spp. The abundance of *Lactobacillus* spp. was maintained at <6% throughout the 6 months in F1 DS silage, regardless of BP addition. Likewise, *Streptococcus* spp. were found at 5.3% after ensiling, however, the abundance was almost unchanged throughout ensiling. Meanwhile, the abundance of *Bacillus* spp. increased to 16.7–19.7% at 6 months from <1% at the time of ensiling in F1 DS silage.

*Acinetobacter* spp. were by far the most abundant bacteria (91.9%) in pre-ensiled F2 SCR, followed by *Enhydrobacter* spp. (4.47%). In F2 PS silage, the abundance of *Acinetobacter* spp. was <10% at 3 months, and increased to >20% at 6 months, regardless of BP addition. Similar to pre-ensiled F1 SCR, although *Lactobacillus* spp. were initially undetectable at ensiling in F2 PS silage, the abundance increased to 7.19–16.6% at 2 weeks and 32.5–50.7% at 3 months. *Streptococcus* spp. were observed at <0.5% at ensiling, >20% at 2 weeks, and 8.5–18.0% at 6 months in F2 PS silage. As a result of long-term storage, *Bacillus* (1.7–2.1%) and *Enterococcus* spp. (4.0–5.6%) were also observed in F2 PS silage. Unlike the F1 SCR silages, *Clostridium* spp. were found at around <1.0% in F2 PS silage stored for 6 months.

Keeping SCR piled and unprocessed for 2 days decreased the abundance of *Acinetobacter* spp. (69.2%) in pre-ensiled F2 SCR. Although *Acinetobacter* spp. remained the most abundant taxon at 2 weeks, *Bacillus* spp. became apparent after prolonged ensiling and were the most abundant bacteria (46.8–65.3%) at 6 months in F2 DS silage. The abundance of *Lactobacillus* spp. in F2 DS silage remained low (<10%), regardless of BP addition.

**Figure 2:**
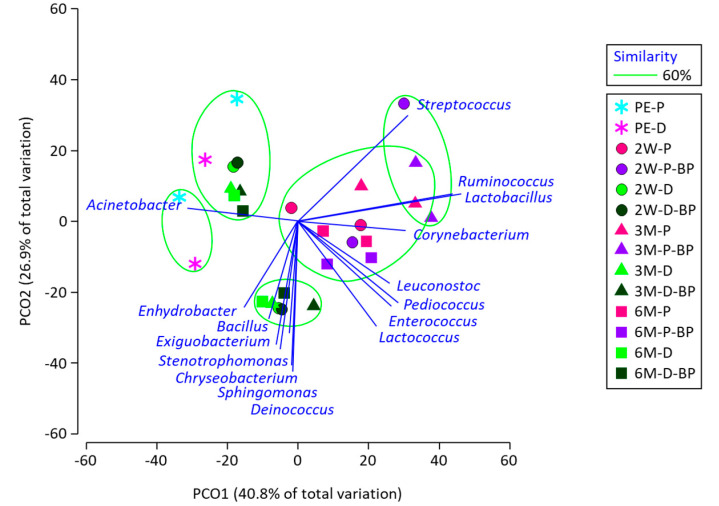
Principal coordinates plot characterizing bacterial microbiota of soybean curd residue silage prepared with prompt and delayed sealing and with and without beet pulp addition. The operational taxonomy unit with a Pearson’s correlation of >0.7 is overlaid on the plot as vectors. Samples enclosed in the same group at a 60% similarity level are denoted with green circles. PE, 2W, 3M, and 6M indicate pre-ensiled material and silage stored for 2 weeks, 3 months, and 6 months, respectively. P, D, and BP after the hyphenation denote prompt sealing, delayed sealing, and beet pulp addition, respectively.

The PCoA results demonstrated that the microbiota of SCR silage was clearly separated by sealing time (Figure 2). Although the difference between F1 and F2 was not observed for PS silage, the difference was clear for DS silage. The effect of BP addition on silage microbiota was not distinctive in either the PS or DS silages.

### 3.3. Fungal Microbiota

**Figure 3:**
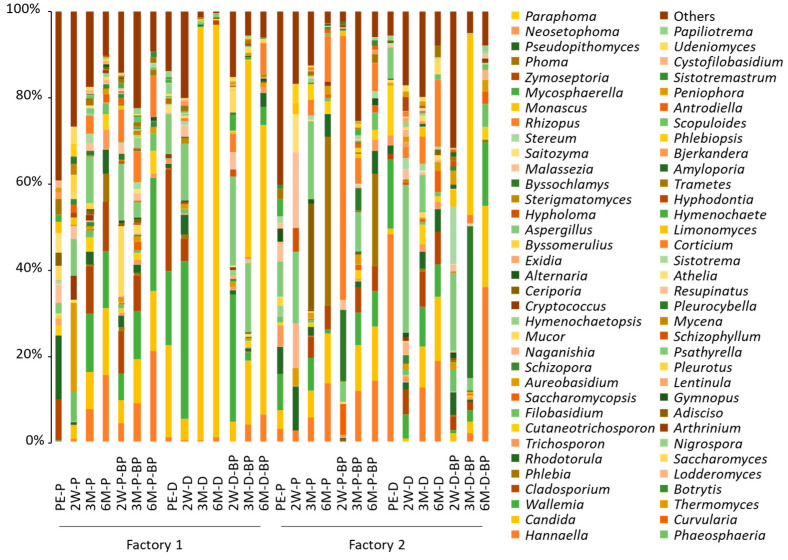
Genus-level fungal microbiota in soybean curd residue silage prepared with prompt and delayed sealing and with and without beet pulp addition. PE, 2W, 3M, and 6M indicate pre-ensiled material and silage stored for 2 weeks, 3 months, and 6 months, respectively. P, D, and BP after the hyphenation denote prompt sealing, delayed sealing, and beet pulp addition, respectively.

The number of non-chimeric sequence reads was low for the analysis of fungal microbiota, with an average of 7649 and 5854 for F1 and F2 samples, respectively. However, diverse fungal species with relative abundances of <10%, except for *Rhodotorula* spp. (14.8%), were found in pre-ensiled F1 SCR (Figure 3). In F1 PS silage, *Aspergillus*, *Candida*, *Cladosporium*, *Aureobasidium*, *Hannaella*, *Wallemia*, and *Mucor* spp. were detected at >10% at 2 weeks. The major fungi detected at 3 months were *Aspergillus* (3.6–11.0%), *Cladosporium* (8.3–11.1%), and *Wallemia* spp. (11.1–13.6%) at 3 months, and *Candida* (14.0–15.4%), *Hannaella* (15.6–21.2%), and *Wallemia* spp. (13.4–26.3%) at 6 months in F1 PS silage.

After SCR was piled and unprocessed for 2 days, *Candida* (21.7%), *Cladosporium* (23.6%), and *Wallemia* spp. (17.2%) were found to be major fungi, while the abundance of *Rhodotorula* spp. was substantially low (1.1%) in pre-ensiled F1 SCR. *Wallemia* (17.2–29.7%) and *Aspergillus* spp. (13.1–20.9%) were found in F1 DS silage at 2 weeks, while *Candida* spp. became predominant (>95%) at 3 months. The abundance of *Candida* spp. (67%) was also high at 6 months in F2 DS silage stored with BP.

Fungal microbiota were also highly diverse in pre-ensiled F2 SCR. Although *Aspergillus*, *Rhodotorula*, and *Wallemia* spp. were the major species, their abundance was only <10%. *Candida* (6.1–12.8%) and *Hannaella* spp. (5.9–14.2%) became apparent at 3 and 6 months in F2 PS silage, respectively. *Phlebia* spp. was detected at <0.2% at 3 months, but its abundance increased abruptly (21.4–39.4%) in F2 PS silage at 6 months.

*Hannaella* (48.1%), *Wallemia* (16.3%), and *Cutaneotrichosporon* spp. (11.5%) were the major fungi in pre-ensiled F2 SCR after being piled and unprocessed for 2 days. The abundances of *Hannaella* and *Wallemia* spp. decreased at 2 weeks and 3 months but returned to high levels (18.8–36.2%) at 6 months in F2 DS silage. The abundance of *Cutaneotrichosporon* spp. declined to <3% at 6 months. Although *Aspergillus* spp. were found at high levels (18.3–34.0%) at 2 weeks, the species was not observed in F2 DS silage at 6 months. The abundance of *Candida* spp. gradually increased during ensiling, culminating in an abundance of 15.0–18.7% at 6 months in F2 DS silage. *Byssochlamys* (35.2%) and *Monascus* spp. (42.0%) were the major fungi in F2 DS silage at 3 months. However, these fungi became undetectable at 6 months in F2 DS silage stored with BP.

**Figure 4:**
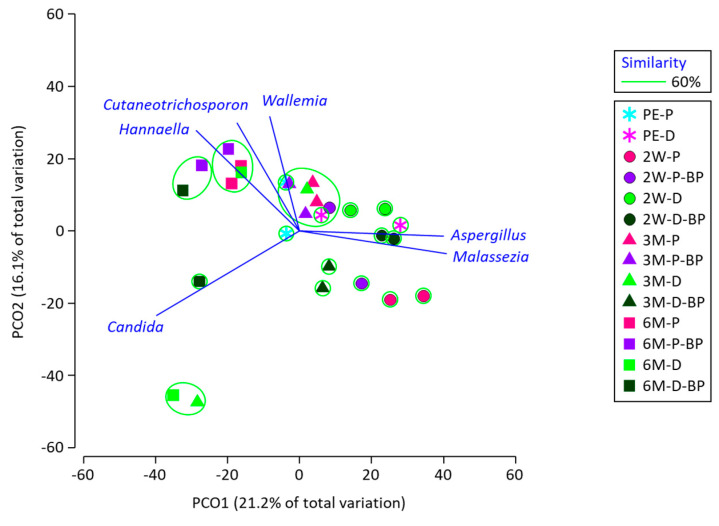
Principal coordinates plot characterizing fungal microbiota of soybean curd residue silage prepared with prompt and delayed sealing and with and without beet pulp addition. The operational taxonomy unit with a Pearson’s correlation of >0.7 is overlaid on the plot as vectors. Samples enclosed in the same group at a 60% similarity level are denoted with green circles. PE, 2W, 3M, and 6M indicate pre-ensiled material and silage stored for 2 weeks, 3 months, and 6 months, respectively. P, D, and BP after the hyphenation denote prompt sealing, delayed sealing, and beet pulp addition, respectively.

The PCoA results illustrate the diversity of fungal microbiota in SCR (Figure 4). All data for silages at 2 weeks were grouped separately. Silages at 6 months were characterized by *Cutaneotrichosporon* and *Hannaella* spp., but no fungi showed differences between F1 and F2 and the effect of BP addition.

## 4. Discussion

Tofu is a traditional Asian food, and many studies have been conducted to examine the process of soybean fermentation and identify additives for SCR ensiling [4]. Because of its milled and mashed physical properties, SCR is regarded as an easy material to ensile. As a result, the fermentation of this legume can be performed by lactic acid without the need for additives. The microbiota associated with SCR ensiling have also been examined in a number of studies [10]. However, except for the total mixed ration silage, containing SCR as an ingredient, most of the previous findings have been derived from plate culture, which enumerated limited taxa [11,12]. The rancidity arising from piling and unprocessing is an important obstacle to the widespread use of SCR. Although this study examined two types of wet SCRs derived from two different factories (F1 and F2), the microbiota associated with SCR ensiling were found to be different from those associated with forage ensiling.

After separation from soy milk, wet SCR (pre-ensiled PS material) had small amounts (16.7–22.1 g/kg DM in total) of soluble sugars, with sucrose and stachyose being the major sugars. In pre-ensiled DS materials, the sucrose content decreased, stachyose disappeared, and maltose appeared, indicating that the microbiota of wet SCR exerted starch hydrolyzing activity before ensiling. *Acinetobacter* spp. were the most abundant bacteria in pre-ensiled SCR. The fact that *Acinetobacter* spp. possess adequate levels of amylase activity [13] supports this. Of note, stachyose and raffinose are soy oligosaccharides that can promote the growth of lactic acid bacteria [14]; hence, the difference in the soy oligosaccharides content between PS and DS pre-ensiled SCR could account for a higher abundance of *Lactobacillus* spp. in PS than that in DS silages.

A diverse range of genera, including *Agrobacterium*, *Erwinia*, *Methylobacterium*, *Microbacterium*, *Pedobacter*, *Pseudomonas*, and *Sphingomonas* spp., are the most abundant species in forage ensiling [15]. *Acinetobacter* spp. (*Moraxellaceae*) are regarded as minor bacterial species in pre-ensiled forages. However, in a previous study, *Acinetobacter* spp. was detected at a high abundance in pre-ensiled napier grass and sudan grass [16]. *Acinetobacter* spp. are aerobic, non-fermentative, and ubiquitous in nature [17]; hence, because of the preceding manufacturing processes, including heating, pre-ensiled SCR was likely to be vulnerable to contamination by factory-associated microbiota. In F2 SCR, the levels of *Enhydrobacter* spp. numerically increased during piling and unprocessing for 2 days. However, starch hydrolysis activity has not yet been observed in *Enhydrobacter* spp.

Our finding that lactic acid dominated the fermentation of SCR silage with PS is in agreement with other studies [4,10]. Although the soluble sugar content was low, the lactic acid content reached >30 g/kg DM after 2 weeks. Although the initial abundances of lactic acid-producing bacteria were quite small, *Lactobacillus* and *Streptococcus* spp. were the main bacteria in F1 PS silage, while *Lactobacillus*, *Streptococcus*, *Enterococcus*, and *Pediococcus* spp. were the main bacteria in F2 PS silage. Based on the PCoA results, the bacterial microbiota of PS silage was found to set up and remain separated from that of DS silage. Interestingly, although *Acinetobacter* and *Bacillus* spp. remained the most abundant bacteria during ensiling, lactic acid was prevalent in DS silage. Since *Acinetobacter* spp. lack the ability to produce lactic acid, *Bacillus* spp. could have been involved in lactic acid fermentation in DS silage. Although *Bacillus* spp. are generally regarded as strictly aerobic bacteria, facultative anaerobic species are also present, and their ability to produce lactic acid has been acknowledged [18,19,20].

*Bacillus* spp. are known to grow well in soybean products, exhibiting various enzyme activities, including protease, amylase, cellulase, and pectinase [21,22]. In the present study, when SCR was unprocessed and unsealed for 2 days, the abundance of *Bacillus* spp. increased from 0.03–0.05% to 0.49–0.90%, while maltose became detectable, and a substantial amount of NH_3_–N (0.64–1.37 g/kg DM) was produced. *Paenibacillus*, *Exiguobacterium*, *Enhydrobacter*, *Stenotrophomonas*, and *Deinococcus* spp. also increased their abundance during piling and unprocessing for 2 days. Further, *Acinetobacter* spp., the most abundant bacteria in pre-ensiled SCR, were also shown to exert both proteinase and deaminase activities [23]. Hence, the intensive production of NH_3_–N observed in the DS materials could be attributed to the collective activities of bacteria, including *Bacillus* and *Acinetobacter* spp. The NH_3_–N content of PS silage without added BP was as low as 0.31 g/kg DM, indicating that the proteolysis and deamination activities were efficiently suppressed under PS conditions.

Although the cow gut microbiota has been demonstrated to be robust and not easily affected by silage-derived microbiota, several lactic acid bacteria species were shown to inhabit both silage and feces [24]. Moreover, *Bacillus* spp., isolated from fermented soybean, were proven to improve milk production and promote the growth of total, proteolytic, and amylolytic bacteria in the rumen [25]. Therefore, the potential of SCR silage as a vehicle for probiotics delivery deserves to be explored.

The fungal microbiota in SCR was found to be diverse, with no distinctive changes after the fermentation of the PS and DS silages. These results suggest that bacterial microbiota play a key role in the fermentation of SCR silage. Although *Candida*, *Aspergillus*, *Cladosporium*, *Hannaella*, and *Wallemia* spp. were the major fungal species in the PS and DS silages, information on *Cladosporium*, *Hannaella*, and *Wallemia* spp. is scarce in relation to their use in feed preservation. Although Pascal et al. [26] found *Hannaella* spp. in whole crop corn silage at a relative abundance of around 20%, their roles in anaerobic storage and spoilage are not known. *Wallemia* and *Cladosporium* spp. are considered spoilage fungi [27,28]; however, no studies have been conducted on the detection and isolation of these species from silage. Furthermore, microbiota assessment has so far been performed mainly on bacteria in silage research. Thus, further studies will be needed to improve our understanding of microbiota control.

## 5. Conclusions

Although *Acinetobacter* spp. were exclusively found in wet SCR at the time of generation in the tofu factories, predominant lactic acid fermentation was obtained in SCR silage by low levels of *Lactobacillus* spp., *Streptococcus* spp., and *Pediococcus* spp. Further, *Bacillus* spp. were involved in lactic acid production in DS silage and long-stored PS silage. Fungal microbiota were highly diverse and no specific genera were associated with factory, sealing time, or fermentation products; hence, bacterial microbiota played a key role in the fermentation of SCR silage. The rancidity arising from piling and unprocessing in wet SCR could be attributed to the collective activities of bacteria, including *Bacillus* spp. and *Acinetobacter* spp. Owing to preceding processing, including heating, distinctive microbiota may have participated in the ensiling of wet by-products.

## Figures and Tables

**Table 1:** Chemical composition and culturable microbiota count of soybean curd residue (SCR) used for ensiling study with prompt and delayed sealing.

Item	Factory 1 SCR		Factory 2 SCR	
	Prompt Sealing	Delayed Sealing	Prompt Sealing	Delayed Sealing
Dry matter (g/kg)	182	194	239	232
Fructose (g/kg DM)	2.43	3.85	0.89	1.48
Glucose (g/kg DM)	2.62	0.97	0.39	1.89
Sucrose (g/kg DM)	5.70	3.11	14.3	1.92
Maltose (g/kg DM)	nd	1.28	nd	0.88
Raffinose (g/kg DM)	1.40	1.85	1.25	0.95
Stachyose (g/kg DM)	4.53	0.00	5.32	0.00
pH	7.21	6.50	7.33	7.00
Lactic acid (g/kg DM)	5.07	15.7	0.28	1.93
Acetic acid (g/kg DM)	nd	2.12	nd	1.22
NH_3_-N (g/kg DM)	nd	1.37	nd	0.64
Lactic acid bacteria (log cfu/g)	7.21	8.92	5.81	7.63
Yeast and mold (log cfu/g)	<2.0	8.41	5.06	7.89

nd; not detected.

**Table 2:** Composition of fermentation products of factory 1 soybean curd residue silage prepared with prompt and delayed sealing (S) and with and without beet pulp (BP) addition.

Item		Prompt Sealing		Delayed Sealing			Two-Way ANOVA		
		Control	+BP	Control	+BP	SE	S	BP	S × BP
pH	2W	4.39	4.18	4.28	4.32	0.08	NS	NS	NS
	3M	4.16	4.20	4.24	4.09	0.02	NS	*	**
	6M	4.12	4.08	4.22	4.08	0.02	NS	**	NS
Lactic acid (g/kg DM)	2W	37.5	33.9	55.5	41.2	1.02	**	**	**
	3M	48.2	39.6	79.8	53.8	1.79	**	**	**
	6M	54.5	39.1	75.6	50.7	2.12	**	**	NS
Acetic acid (g/kg DM)	2W	2.65	3.98	7.04	6.94	0.37	**	NS	NS
	3M	4.45	8.16	14.9	13.1	0.80	**	NS	**
	6M	7.69	10.7	12.6	10.1	0.70	*	NS	**
Propionic + Butyric	2W	0.00	0.00	0.00	0.00	0.00	NS	NS	NS
acids (g/kg DM)	3M	0.00	0.00	0.00	0.00	0.00	NS	NS	NS
	6M	0.00	0.00	2.08	0.00	0.42	*	*	*
Ethanol (g/kg DM)	2W	2.33	3.24	4.04	3.67	0.25	**	NS	*
	3M	1.32	3.19	2.61	1.97	0.25	NS	*	**
	6M	0.00	0.00	3.68	2.79	0.17	**	*	*
NH_3_-N (g/kg DM)	2W	0.06	0.10	1.98	1.35	0.12	**	*	*
	3M	0.13	0.12	1.08	0.48	0.09	**	**	**
	6M	0.13	0.07	2.05	0.83	0.17	**	**	*

2W; 2 weeks, 3M; 3 months, 6M; 6 months. SE; pooled standard error. NS; not significant, *; *p* < 0.05, ** *p* < 0.01.

**Table 3:** Composition of fermentation products of factory 2 soybean curd residue silage prepared with prompt and delayed sealing (S) and with and without beet pulp (BP) addition.

Item		Prompt Sealing		Delayed Sealing			Two-Way ANOVA		
		Control	+BP	Control	+BP	SE	S	BP	S × BP
pH	2W	4.38	4.26	4.19	4.05	0.02	**	**	NS
	3M	4.31	4.25	4.14	4.01	0.04	**	*	NS
	6M	4.36	4.15	4.12	3.99	0.03	**	**	NS
Lactic acid (g/kg DM)	2W	35.5	29.3	78.7	53.3	1.48	**	**	**
	3M	50.9	42.0	88.5	61.8	1.95	**	**	**
	6M	42.9	34.4	78.9	54.3	2.35	**	**	**
Acetic acid (g/kg DM)	2W	2.78	3.74	16.4	7.51	0.38	**	**	**
	3M	7.68	6.87	24.7	13.6	0.92	**	**	**
	6M	4.91	6.62	18.4	10.5	0.81	**	**	**
Propionic + Butyric	2W	0.00	0.00	0.00	0.00	0.00	NS	NS	NS
acids (g/kg DM)	3M	2.52	0.87	0.00	0.00	0.12	**	**	**
	6M	21.7	17.2	3.38	1.91	0.80	**	**	NS
Ethanol (g/kg DM)	2W	0.73	2.08	2.20	0.96	0.30	NS	NS	**
	3M	0.00	2.65	0.00	0.00	0.03	**	**	**
	6M	0.76	0.45	0.00	0.00	0.23	*	NS	NS
NH_3_-N (g/kg DM)	2W	0.15	0.13	2.05	1.20	0.11	**	**	**
	3M	0.20	0.13	1.45	0.73	0.05	**	**	**
	6M	0.31	0.11	2.05	1.09	0.10	**	**	**

2W; 2 weeks, 3M; 3 months, 6M; 6 months. SE; pooled standard error. NS; not significant, *; *p* < 0.05, **; *p* < 0.01.

## References

[1] Orosz S., Davies D.R., Daniel J.L.P., Morais G., Junges D., Nussio L.G. (2015). Short and long term storage of wet by-products fed by ruminants. Proceeding of the 17th International Silage Conference, Piracicaba, Brazil, 1–3 July 2015.

[2] Knoblich M., Anderson B., Latshaw D. (2005). Analyses of tomato peel and seed byproducts and their use as a source of carotenoids. J. Sci. Food Agric..

[3] Kamble D.B., Rani S. (2019). Bioactive components, in vitro digestibility, microstructure and application of soybean residue (okara): A review. Legume Sci..

[4] Amaha K., Sasaki Y., Segawa T. (1999). Utilization of Tofu (Soybean Curd) by-Products as Feed for Cattle. http://www.agnet.org/htmlarea_file/library/20110716100439/eb419.pdf.

[5] Tran T.M.T., Nguyen H.V., Nishino N. (2014). A pilot examination of the fermentation products, aerobic stability and bacterial community of total mixed ration silage produced in Vietnam. Grassl. Sci..

[6] Nishino N., Harada H., Sakaguchi E. (2003). Evaluation of fermentation and aerobic stability of wet brewers’ grains ensiled alone or in combination with various feeds as a total mixed ration. J. Sci. Food Agric..

[7] Yu Z., Morrison M. (2014). Improved extraction of PCR-quality community DNA from digesta and fecal samples. Biotechniques.

[8] Nelson M.C., Morrison H.G., Benjamino J., Grim S.L., Graf J. (2014). Analysis, optimization and verification of Illumina-generated 16S rRNA gene amplicon surveys. PLoS ONE.

[9] Ihrmark K., Bödeker I.T.M., Cruz-Martinez K., Friberg H., Kubartova A., Schenck J., Strid Y., Stenlid Y., Brandström-Durling M., Clemmensen K.E. (2012). New primers to amplify the fungal ITS2 region—Evaluation by 454-sequencing of artificial and natural communities. FEMS Microbiol. Ecol..

[10] Wang F., Nishino N. (2008). Ensiling of soybean curd residue and wet brewers grains with or without other feeds as a total mixed ration. J. Dairy Sci..

[11] Hiwatashi M., Kano S., Kato T. (2015). Development of a preservation method for okara using lactic acid fermentation. Nippon Shokuhin Kagaku Kogaku Kaishi.

[12] Li Y., Wang F., Nishino N. (2016). Lactic acid bacteria in total mixed ration silage containing soybean curd residue: Their isolation, identification and ability to inhibit aerobic deterioration. Asian Australas. J. Anim. Sci..

[13] Onishi H., Hidaka O. (1978). Purification and properties of amylase produced by a moderately halophilic *Acinetobacter* sp.. Can. J. Microbiol..

[14] Bordignon J.R., Nakahara K., Yoshihashi T., Nikkuni S. (2004). Hydrolysis of isoflavones and consumption of oligosaccharides during lactic acid fermentation of soybean milk. JARQ.

[15] Ni K., Tang T.M., Tran T.M.T., Tsuruta T., Pang H., Nishino N. (2017). Comparative microbiota assessment of wilted Italian ryegrass, whole crop corn, and wilted alfalfa silage using denaturing gradient gel electrophoresis and next-generation sequencing. Appl. Microbiol. Biotechnol..

[16] Nazar M., Wang S., Zhao J., Dong Z., Li J., Kara N.A., Shao T. (2020). The feasibility and effects of exogenous epiphytic microbiota on the fermentation quality and microbial community dynamics of whole crop corn. Bioresour. Technol..

[17] Doughari H.J., Ndakidemi P.A., Human I.S., Benade S. (2011). The ecology, biology and pathogenesis of *Acinetobacter* spp.: An overview. Microbes Environ..

[18] Ohara H., Yahata M. (1996). L-Lactic acid production by *Bacillus* sp. in anaerobic and aerobic culture. J. Ferment. Bioeng..

[19] Payot T., Chemaly Z., Fick M. (1999). Lactic acid production by *Bacillus coagulans*-Kinetic studies and optimization of culture medium for batch and continuous fermentations. Enzym. Microb. Technol..

[20] Lara E.C., Basso F.C., de Assis F.B., Souza F.A., Berchielli T.T., Reis R.A. (2016). Changes in the nutritive value and aerobic stability of corn silages inoculated with *Bacillus subtilis* alone or combined with *Lactobacillus plantarum*. Anim. Prod. Sci..

[21] Sarker P.K., Cook P.E., Owens J.D. (1993). *Bacillus* fermentation of soybeans. World J. Microbiol. Biotechnol..

[22] Chen T., Wang M., Jiang S., Xiong S., Zhu D., Wei H. (2011). Investigation of the microbial changes during koji-making process of Douchi by culture-dependent techniques and PCR-DGGE. Int. J. Food Sci. Technol..

[23] Ashwini M.S., Shilpa S.M., Smita B.P., Balu A.C. (2015). Isolation, biotyping, biochemical and physiological characterization of marine *Acinetobacter* isolated from west coast of India. Int. J. Curr. Microbiol. App. Sci..

[24] Han H., Ogata Y., Yamamoto T., Nagao S., Nishino N. (2014). Identification of lactic acid bacteria in the rumen and feces of dairy cows fed total mixed ration silage to assess the survival of silage bacteria in the gut. J. Dairy Sci..

[25] Sun P., Wang J.Q., Deng L.F. (2013). Effects of *Bacillus subtilis natto* on milk production, rumen fermentation and ruminal microbiome of dairy cows. Animal.

[26] Pascal D., Julien T., Frederique C.D. (2019). Dynamic succession of microbiota during ensiling of whole plant corn following inoculation with *Lactobacillus buchneri* and *Lactobacillus hilgardii* alone or in combination. Microorganisms.

[27] Janja Z., Nina G.C. (2018). The genus *Wallemia*-from contamination of food to health threat. Microorganisms.

[28] Peter G., Mounier J., Garnier L., Soos D., Dlauchy D. (2019). *Cutaneotrichosporon suis* sp. nov., a lipolytic yeast species from food and food-related environment. Int. J. Syst. Evol. Microbiol..

